# 1408. Population-based Nontuberculous Mycobacteria Surveillance in Four Emerging Infections Program Sites, October 2019–March 2020

**DOI:** 10.1093/ofid/ofab466.1600

**Published:** 2021-12-04

**Authors:** Kelly A Jackson, Devra Barter, Christopher A Czaja, Helen Johnston, Ruth Lynfield, Ruth Lynfield, Paula Snippes Vagnone, Laura Tourdot, Nancy Spina, Ghinwa Dumyati, Shantel Peters, Gabriela Escutia, Rebecca Pierce, Emily Henkle, D Rebecca Prevots, Max Salfinger, Kevin L Winthrop, Kevin L Winthrop, Nadege Charles Toney, Shelley Magill, Cheri Grigg

**Affiliations:** 1 Centers for Disease Control and Prevention, Atlanta, GA; 2 Colorado Department of Public Health and Environment, Denver, Colorado; 3 Minnesota Department of Health, St. Paul, MN; 4 Minnesota Department of Health Laboratory, St. Paul, MN; 5 New York State Department of Health, Albany, NY; 6 New York Rochester Emerging Infections Program at the University of Rochester Medical Center, Rochester, NY; 7 University of Rochester Medical Center, Rochester, New York; 8 Oregon Health Authority, Portland, Oregon; 9 Oregon Health & Science University, Portland, OR; 10 National Institutes of Health, National Institute of Allergy and Infectious Diseases, Bethesda, Maryland; 11 University of South Florida College of Public Health, Tampa, Florida; 12 Division of Healthcare Quality Promotion, Centers for Disease Control and Prevention, Atlanta, Georgia

## Abstract

**Background:**

Nontuberculous mycobacteria (NTM) cause pulmonary (PNTM) and extrapulmonary (ENTM) disease. NTM infections are difficult to diagnose and treat; environmental exposures occur in both healthcare and community settings. Few population-based studies describe NTM disease epidemiology. Current data indicate PNTM disease and ENTM skin and soft tissue infections are increasing. We describe findings from a multi-site pilot of population-based NTM surveillance.

**Methods:**

CDC’s Emerging Infections Program conducted active, laboratory- and population-based surveillance for NTM cases occurring in 4 sites (Colorado [5 counties], Minnesota [2 counties], New York [2 counties], and Oregon [3 counties PNTM; statewide ENTM]) during October 1, 2019–March 31, 2020. PNTM cases were defined according to current published microbiologic criteria, based on isolation of NTM in respiratory cultures or tissue. ENTM cases required NTM isolation from a non-pulmonary specimen, excluding stool or rectal swabs. Demographic, clinical, exposure, and laboratory data were collected via medical record review. We calculated overall incidence per 100,000 population using census data and performed descriptive analyses of medical record data.

**Results:**

Overall, 299 NTM cases were reported (231 [77%] PNTM); *M. avium* was the most commonly isolated species (Table). NTM incidence was 3.8 per 100,000 (PNTM 3.1/100,000; ENTM 0.7/100,000). Most patients with available data had ≥1 sign or symptom in the 14 days before culture (63 [97%] ENTM, 203 [92%] PNTM). During the surveillance period, 187 (63%) had their first infection-defining culture collected in an outpatient setting (33 [49%] ENTM, 154 [67%] PNTM). Of PNTM cases, 145 (64%) were female, and 154 (67%) had underlying pulmonary disease. Among ENTM cases, 29 (43%) were female, 9 (13%) had diabetes, 8 (12%) had HIV and 27 (40%) had infection at the site of a medical device or healthcare procedure. Common ENTM infection types were lymphadenitis (16 [24%]) and skin abscess (12 [18%]).

Table. Characteristics of persons with NTM infection identified in population-based surveillance, October 1, 2019–March 31, 2020.

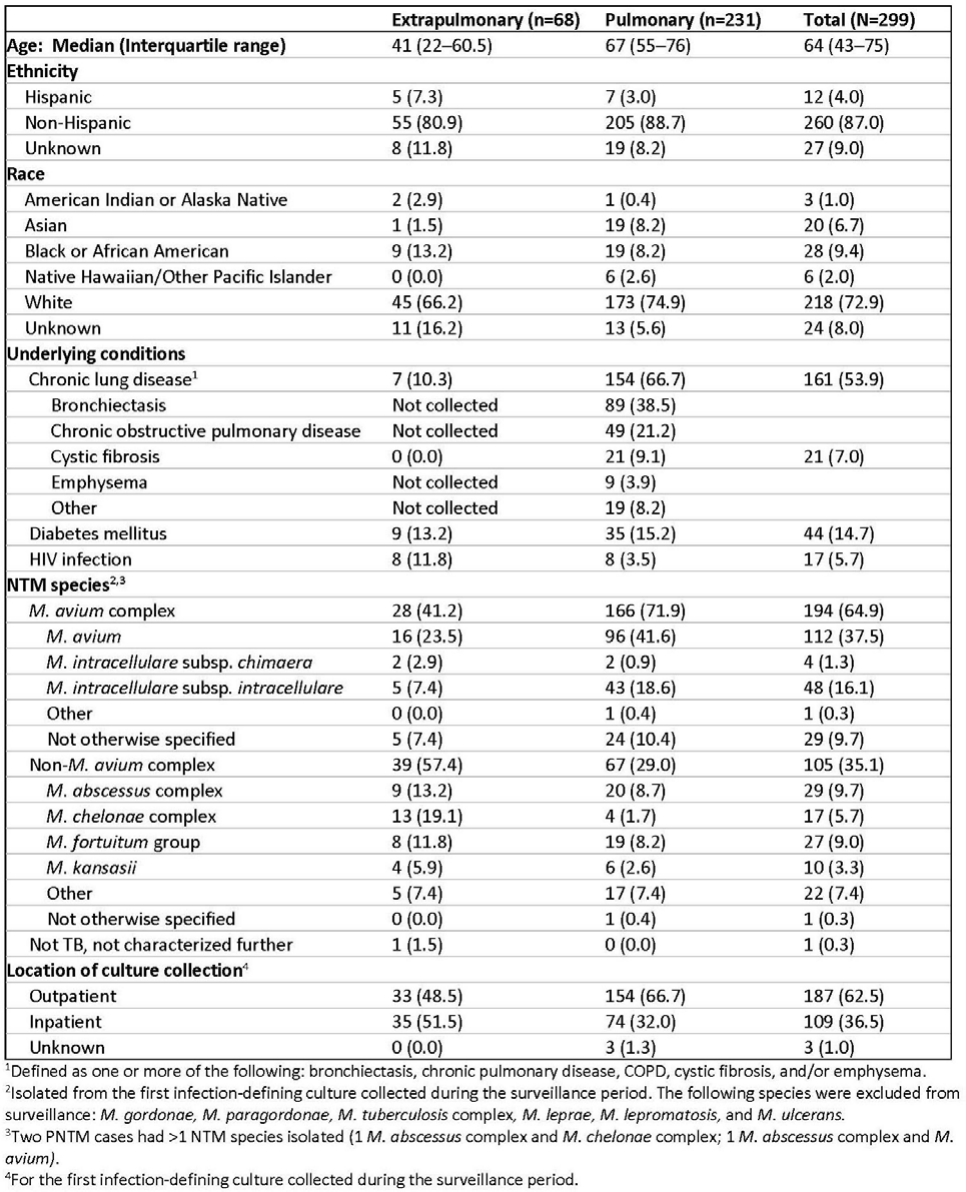

**Conclusion:**

Characterizing disease burden and affected populations with population-based NTM surveillance will provide data to inform potential interventions and monitor prevention strategy impact.

**Disclosures:**

**Christopher A. Czaja, MD, DrPH**, **Centers for Disease Control and Prevention** (Grant/Research Support) **Ruth Lynfield, MD**, Nothing to disclose **Ghinwa Dumyati, MD**, **Pfizer** (Grant/Research Support)**Roche Diagnostics** (Advisor or Review Panel member) **Emily Henkle, PhD, MPH**, **AN2** (Consultant, Advisor or Review Panel member)**Zambon** (Advisor or Review Panel member) **Kevin L. Winthrop, MD, MPH**, **Insmed** (Consultant, Grant/Research Support)**Paratek** (Consultant)**RedHill** (Consultant)**Spero** (Consultant) **Kevin L. Winthrop, MD, MPH**, **Insmed** (Consultant, Research Grant or Support)**Paratek** (Consultant)**RedHill Biopharma** (Consultant)**Spero** (Consultant)

